# Agreement Between Self-reports and Photos to Assess e-Cigarette Device and Liquid Characteristics in Wave 1 of the Vaping and Patterns of e-Cigarette Use Research Study: Web-Based Longitudinal Cohort Study

**DOI:** 10.2196/33656

**Published:** 2022-04-27

**Authors:** Elizabeth Crespi, Jeffrey J Hardesty, Qinghua Nian, Joshua Sinamo, Kevin Welding, Ryan David Kennedy, Joanna E Cohen

**Affiliations:** 1 Institute for Global Tobacco Control Department of Health, Behavior & Society Johns Hopkins Bloomberg School of Public Health Baltimore, MD United States

**Keywords:** tobacco, e-cigarette, methodology, internet, photo, survey, self-report

## Abstract

**Background:**

e-Cigarette device and liquid characteristics are highly customizable; these characteristics impact nicotine delivery and exposure to toxic constituents. It is critical to understand optimal methods for measuring these characteristics to accurately assess their impacts on user behavior and health.

**Objective:**

To inform future survey development, we assessed the agreement between responses from survey participants (self-reports) and photos uploaded by participants and the quantity of usable data derived from each approach.

**Methods:**

Adult regular e-cigarette users (≥5 days per week) aged ≥21 years (N=1209) were asked questions about and submitted photos of their most used e-cigarette device (1209/1209, 100%) and liquid (1132/1209, 93.63%). Device variables assessed included brand, model, reusability, refillability, display, and adjustable power. Liquid variables included brand, flavor, nicotine concentration, nicotine formulation, and bottle size. For each variable, percentage agreement was calculated where self-report and photo data were available. Krippendorff *α* and intraclass correlation coefficient (ICC) were calculated for categorical and continuous variables, respectively. Results were stratified by device (disposable, reusable with disposable pods or cartridges, and reusable with refillable pods, cartridges, or tanks) and liquid (customized and noncustomized) type. The sample size for each calculation ranged from 3.89% (47/1209; model of disposable devices) to 95.12% (1150/1209; device reusability).

**Results:**

Percentage agreement between photos and self-reports was substantial to very high across device and liquid types for all variables except nicotine concentration. These results are consistent with Krippendorff *α* calculations, except where prevalence bias was suspected. ICC results for nicotine concentration and bottle size were lower than percentage agreement, likely because ICC accounts for the level of disagreement between values. Agreement varied by device and liquid type. For example, percentage agreement for device brand was higher among users of reusable devices (94%) than among users of disposable devices (75%). Low percentage agreement may result from poor participant knowledge of characteristics, user modifications of devices inconsistent with manufacturer-intended use, inaccurate or incomplete information on websites, or photo submissions that are not a participant’s most used device or liquid. The number of excluded values (eg, self-report was “don’t know” or no photo submitted) differed between self-reports and photos; for questions asked to participants, self-reports had more usable data than photos for all variables except device model and nicotine formulation.

**Conclusions:**

Photos and self-reports yield data of similar accuracy for most variables assessed in this study: device brand, device model, reusability, adjustable power, display, refillability, liquid brand, flavor, and bottle size. Self-reports provided more data for all variables except device model and nicotine formulation. Using these approaches simultaneously may optimize data quantity and quality. Future research should examine how to assess nicotine concentration and variables not included in this study (eg, wattage and resistance) and the resource requirements of these approaches.

## Introduction

e-Cigarettes are devices that heat a liquid to produce an aerosol for inhalation. The device itself typically consists of a battery, an electrical heater (eg, an atomizer or coil), a container (eg, cartridge, pod, or tank) to hold liquid, and a mouthpiece for inhalation [1,2]. e-Cigarette liquids often comprise nicotine, propylene glycol, vegetable glycerin, and flavorants, but can also contain tetrahydrocannabinol, cannabidiol, and vitamins [1,3-5]. e-Cigarette devices and liquids are highly customizable; for example, adjustable power and modifiable coils allow users to alter their device and its settings, and users can mix flavors in various combinations and proportions [5,6]. In 2014, users were able to select from >450 e-cigarette device brands available for sale on the internet [7]. More recent research suggests that a similar number of device brands existed in 2017. Although 178 of the device brands for sale in 2014 had ceased operation as of 2017, many were replaced by newly emerged brands [8]. In the same time frame, the number of unique flavors increased from 7700 [7] to >15,500 [8]. In addition, the number of websites selling refillable modifiable devices increased from 117 to 190 between 2014 and 2017, whereas the number of websites selling disposable cig-a-like devices (ie, devices that look similar to cigarettes) decreased by approximately 50 in the same period [8]. This demonstrates the popularity of customizable devices in the market and the quick evolution of the e-cigarette device and liquid market.

Various constituents that may be associated with negative health outcomes have been found in e-cigarette liquids and aerosols, including aldehydes, carbonyls, and heavy metals among others [9]. e-Cigarette users may have vastly different experiences and exposures to toxic constituents depending on the duration of vaping sessions [10], e-cigarette liquid contents [10,11], device types [12,13], and device settings [6,14]. Evidence also indicates that these characteristics may be important determinants of nicotine delivery and influence the risk and severity of e-cigarette dependence [5,6]. It is important to monitor the use of these device and liquid characteristics to better understand e-cigarette use behaviors and patterns and their impact on health to inform policy decisions about these products. However, the wide range of products available, their high level of customizability, and the rapidly evolving marketplace presents challenges for measurement.

Self-report data in health research can be subject to several biases (eg, social desirability, recall period, sampling approach, or selective recall) that affect the validity and reliability of the data [15]. Although self-reported measures have been shown to be reliable for assessing cigarette use [16,17], their reliability for assessing e-cigarette use varies depending on the particular measure used (eg, number of days in the past 30 days and sessions per day) [18-20] and their reliability for certain e-cigarette device characteristics, such as voltage and resistance, is insufficient [21]. Thus, there is a need to examine novel methods for assessing e-cigarette device and liquid characteristics, such as submission and coding of photos of e-cigarette devices and liquids, to understand the potential advantages and disadvantages of these data collection methods and whether various e-cigarette device or liquid characteristics warrant different approaches to measurement. This study assessed the agreement between self-report and photo-coded data for certain e-cigarette device and liquid characteristics to better understand the potential challenges and advantages of each approach.

## Methods

### Study Sample and Protocols

Data were from wave 1 (May 2020 to October 2020) of the Vaping and Patterns of E-cigarette Use Research study, which is a US-based longitudinal cohort study following regular vapers (≥5 days per week) aged ≥21 years through a web-based survey about e-cigarette use patterns and behaviors. Participants of wave 1 were recruited via web-based advertisements on Facebook, Instagram, and Craigslist and flyers and business cards distributed by vape shops. Advertisements were posted in 125 US cities selected for their potential to yield respondents who use e-cigarettes (ie, relatively high population).

After clicking an advertisement, participants were directed to a web-based survey hosted by Virginia Commonwealth University’s REDCap (Research Electronic Data Capture; Vanderbilt University), a Health Insurance Portability and Accountability Act–compliant secure web application for building and managing web-based surveys and databases. Before answering any questions, participants reviewed a consent form and certificate of confidentiality and were asked if they would like to continue with the survey. Then, they provided their contact information (ie, full name, phone number, email address, mailing address, and date of birth).

In addition to answering survey questions about their use patterns and behaviors, participants were asked to submit photos of their most used e-cigarette device and their most used liquid for that device. Upon completion, participants received US $10 Amazon gift codes via mail. To exclude potential bot activity, survey responses were reviewed for non-English or Spanish alphabet (0/2813, 0%), data suggesting inattention or very low knowledge of e-cigarettes or liquids (eg, indicating that they are aged 25 years and began vaping at the age of 50 years or self-reporting JUUL as a disposable device; 38/2813, 1.35%), invalid mailing addresses (13/2813, 0.46%), answering the minimum number of questions (this suggests participant may have previous knowledge of survey skip logic; 0/2813, 0%), completing the survey in <5 minutes (24/2813, 0.85%), missing attention check questions (64/2813, 2.28%), straight lining (0/2813, 0%), failed or incomplete identity authentication (eg, did not provide additional information such as utility bill to confirm identity; 765/2813, 27.2%), or multiple survey attempts (162/2813, 5.76%). Participants (464/2813, 16.5%) were also excluded if their device photos were from the internet (as determined by a Google search describing the image or a Google Reverse Image Search), submitted multiple times, not of an e-cigarette device, or taken in a store. JUUL, Vaporesso, and Voopoo were included as examples in the question prompt for device brand, and records suspected to be bots or professional survey takers frequently reported these 3 brands. Therefore, we decided to consider participants who self-reported one of these 3 brands and submitted a photo of a device that did not match the reported brand to be invalid. In all, 0.11% (3/2813) of the records were not reviewed for various reasons (eg, completed survey after the survey closed) and were excluded from the final data set.

Upon completion of the data collection wave, participants were also excluded if they were found to be highly suspicious in a review of suspicious records (71/2813, 2.52%). Records were comprehensively reviewed if they had incentives returned to the sender or contained data issues such as reporting devices intended for cannabis or oils or beginning to use e-cigarettes before they were commercially available in the United States. Records were considered highly suspicious and were excluded if they contained 1 significant data issue (eg, self-reported liquid contains cannabis derivatives) or a combination of multiple data issues (eg, mismatched self-report and photo device or liquid, patterned responses, or self-reported device or liquid characteristics that do not match self-reported device or liquid). A total of 7875 participants completed the screener, of which 4289 (54.46%) participants were eligible. Of the 4289 participants, 2813 (65.59%) participants completed the survey. Of the 2813 participants, 1604 (57.02%) were excluded after implementing the strategies listed above to avoid bots and professional survey takers, resulting in a sample size of 1209 (42.98%). Researchers (EC and JH) used the Google search engine to search text and markings in submitted photos of devices and liquids to identify the device brand and model and liquid brand and flavor by visually matching the submitted photo with Google search results. Then, the device brand and model and liquid brand and flavor were searched on Google to identify, in the following order of priority, manufacturer, academic (ie, journal articles), retail, and review sites for the given device and liquid and record information about key characteristics of the devices and liquids. One site was sufficient for confirming the information for a characteristic; however, up to 3 sites were searched for each characteristic before categorizing the information as missing. If discrepancies were found between sites for a characteristic, information from the site with highest priority was used. If the device brand and model or liquid brand and flavor could not be identified or the information for a particular characteristic could not be found in the photo or on the web, this information was considered missing. An initial round of reliability testing was conducted for this process to ensure high reliability (≥90%) between researchers.

### Ethics Approval

All participants provided informed consent. The institutional review board at the Virginia Commonwealth University (no HM20015004) approved the study protocol. The Johns Hopkins Bloomberg School of Public Health Institutional Review Board (no 9277) approved reliance on the Virginia Commonwealth University Institutional Review Board.

### Measurements

#### Device Brand and Model

Self-reported device brand and model were assessed using the following question: “What is the brand AND model of the device (eg, JUUL, Vaporesso Luxe, Voopoo Drag 2, etc.)?” As participants who self-reported JUUL, Vaporesso, or Voopoo were excluded if their self-reported and photo brand mismatched, these 3 self-reported brands were excluded from all device brand calculations (270/1209, 22.33%). Device brand and model from photos were determined by searching the identifying text or markings in the submitted photo on Google and finding a visual match on the web. For all calculations, device brand and model were assessed individually rather than as a combined variable.

#### Device Reusability

Self-reported reusability of the device was assessed using the following question: “Is the device (1) reusable (ie, you recharge the device when the battery life is low or at 0%) or (2) disposable (ie, you discard entire device when the battery life is low or at 0%)?” For photos, websites obtained from the Google search conducted for each device were reviewed for mentions of the device being disposable or reusable. Then, the records were coded based on the information on the website.

#### Device Refillability

Self-reported refillability of the device was assessed using the following question: “When the device runs out of e-liquid, do you TYPICALLY (1) discard the empty cartridge or pod and replace with a new and unused cartridge or pod prefilled with e-liquid or (2) refill the empty tank/cartridge/pod with e-liquid from a larger container(s) of e-liquid?” Given that disposable devices cannot be refillable, this question was asked only to participants who indicated that their device was reusable. The information on whether the device was refillable or nonrefillable was obtained from photos by searching the device brand and model on Google and extracting details from websites.

#### Visual Representation of Adjustable Settings (Device Display)

Self-reported presence of a visual representation of adjustable settings on the device was assessed using the following question: “Does the device have a VISUAL DISPLAY that allows you to see the wattage or other vape settings?” Response options included “yes” and “no.” This question was asked only to participants who indicated that their device was reusable. For photos, a visual representation of adjustable settings was defined as a display (eg, a screen, small light, or dial) that shows information about settings that can be adjusted by the user; this information was confirmed from websites for the given device.

#### Adjustable Power

Self-reported ability to adjust device power was assessed using the following question: “Does the device have SETTINGS that allow you to modify power or vapor volume?” Response options included “yes” and “no.” This question was asked only to participants who indicated that their device was reusable. For photos, a device was considered to have adjustable power if websites for the given device indicated that the user can customize the wattage or voltage using a dial or button (not by changing the coil or other internal parts).

#### Liquid Type

Liquid type was assessed using participants’ responses to the following question: “Is your most used e-liquid a (1) customized flavor blend-mixed yourself, (2) customized flavor blend-mixed for you by someone else (3) non-customized flavor?” This question was asked only to users of reusable devices with refillable pods, cartridges, or tanks; users of disposable devices or reusable devices with disposable pods or cartridges were presumed to have noncustomized liquids. Agreement between photos and self-reports on liquid type was not assessed; rather, liquid type was used to stratify results by those using customized and noncustomized liquids.

#### Liquid Brand

Self-reported liquid brand was assessed using the following question: “Do you know the brand of the [cartridge or pod (eg, JUUL, blu, VUSE, etc.]/e-liquid container (eg, Naked 100, Beard Vape, Milkman, etc.)]? [If yes] please specify the brand.” As disposable devices do not have separate liquids, this question was not asked to users of disposable devices. Participants who indicated that they use a customized flavor blend were also excluded from this variable as they were not asked about the brand of their liquid. Photos of the liquids were assessed for brand by searching any identifying text or markings in the photo on Google and reviewing websites for a visual match.

#### Liquid Flavor

Self-reported liquid flavor was assessed using the following question: “What is the flavor of the [device’s (for disposable devices)] e-liquid?” Response options included (1) tobacco; (2) tobacco menthol or menthol; (3) mint; (4) a flavor such as fruit, candy, alcohol, coffee, vanilla, or other food/drink; and (5) no flavor. Liquid flavors from photos were identified using flavor descriptions on the website for the given liquid, and then categorized using the e-cigarette liquid flavor wheel developed by Krusemann et al [22]. If a website was unavailable but the flavor was clearly listed on the container, the photo was used to code the flavor. Then, the flavors were grouped to match the survey question on flavors (ie, tobacco, menthol or tobacco menthol, mint, other, or unflavored).

#### Nicotine Concentration (mg/mL)

Self-reported nicotine concentration of the liquid was assessed using the following question: “Do you know how much nicotine is in the [device’s] e-liquid/flavor blend? [If yes] please specify.” Respondents indicated whether they reported the concentration as mg/mL or a percentage. When participants reported nicotine concentrations as a percentage, the reported value was multiplied by 10 to obtain the mg/mL; exceptions include JUUL, which is reported to be 59 mg/mL for their 5% pods and 35 mg/mL for their 3% pods [23]; NJOY Ace, which is reported to be 58 mg/mL for their 5% pods and 28 mg/mL for their 2.4% pods [24]; and NJOY Daily, which is reported to be 69 mg/mL (rich tobacco flavor) or 68 mg/mL (menthol flavor) for their 6% pods and 51 mg/mL for their 4.5% pods [25]. For photos, nicotine concentration (in mg/mL) was extracted from photos of the liquid bottles or from the manufacturer, academic, retail, or review sites. If the nicotine concentration was not mentioned on the container and multiple concentrations were available on the web for the given liquid, concentration was considered missing for that liquid.

#### Nicotine Formulation

Self-reported nicotine formulation of the liquid was assessed using the following question: “Does the [device’s] e-liquid/flavor blend contain nicotine salts?” Response options included “yes” and “no.” For photos, nicotine formulation was coded as either nicotine salt or free-base nicotine. As websites frequently report when a liquid is a salt but fail to report when a liquid is free-base and 27 mg/mL was the highest confirmed free-base nicotine liquid concentration in our sample, liquids with nicotine concentration ≤27 mg/mL, for which formulation could not be found on any website, were considered to be free-base. Liquids (2/1209, 0.17%) with nicotine concentrations >27 mg/mL, with formulation not found on any website (Vuse Solo Chai and Glas pods) were investigated further and considered to be salts because other liquids by the same brand are exclusively salts, YouTube reviewers indicated the liquid is likely a salt, or other similar devices frequently use salts.

#### Liquid Bottle Size (mL)

Self-reported liquid bottle size was assessed using the following question: “Do you know the bottle size (in milliliters) of your most used e-liquid/flavor blend that you last purchased? [If yes] please specify the bottle size (in mL).” This question was asked only to participants who indicated their device was reusable and refillable. For photos, bottle size (in mL) was extracted from photos of the liquid bottles or from the manufacturer, academic, retail, or review sites. If the bottle size was not mentioned on the bottle and multiple sizes were available on the web for the given liquid, bottle size was considered missing for this liquid.

### Statistical Analysis

Percentage agreement, calculated as the number of records for which self-report and photo data were concordant divided by the total number of records with available self-report and photo data, was calculated for the following variables: device brand, device model, device reusability, device refillability, device display, adjustable power, liquid brand, liquid flavor, nicotine concentration, nicotine formulation, and liquid bottle size. As percentage agreement does not account for agreement expected by chance, Krippendorff *α* was also calculated for nominal categorical variables. This method was chosen for its versatility in the number of raters and types of data [26,27]. For continuous variables, intraclass correlation coefficient (ICC) estimates and 95% CIs were also calculated based on a mean rating (*k*=2), absolute-agreement, 2-way mixed effects model [28].

Percentage agreement, Krippendorff *α*, and ICC calculations were also stratified by self-reported liquid type (ie, noncustomized or customized liquid) and self-reported device type (ie, disposable device, reusable device with disposable pods or cartridges, or reusable device with refillable pods, cartridges, or tanks). Calculations were conducted using Microsoft Excel and Stata (version 16.1; StataCorp).

## Results

### Sample Characteristics

Among our sample, 34.33% (415/1209) were aged 21-29 years, 36.15% (437/1209) were aged 30-39 years, 19.35% (234/1209) were aged 40-49 years, and the remaining 10.17% (123/1209) were aged ≥50 years (Table 1). In addition, 53.02% (641/1209) of the participants were female, 44.99% (544/1209) were male, 0.99% (12/1209) of participants selected the “Other” option for gender, and 0.99% (12/1209) of participants selected “prefer not to answer.*”* Most participants were White (919/1209, 76.01%), followed by multiracial (133/1209, 11%), Black (48/1209, 3.97%), “Other” (36/1209, 2.98%), Asian (24/1209, 1.99%), and American Indian/Alaska Native (9/1209, 0.74%) and 1.99% (24/1209) of the participants selected “prefer not to answer.” Most participants (1088/1209, 89.99%) used e-cigarettes 7 days per week. Of the 1209 participants, 713 (58.97%) did not smoke in the last 30 days; the remaining 496 (41.03%) smoked cigarettes ≥1 time in the last 30 days.

**Table 1:** Sociodemographic characteristics of survey participants (N=1209).

Sociodemographic characteristic			Participants, n (%)
**Geographic location**			
	Midwest	216 (17.87)	
	Northeast	130 (10.75)	
	South	491 (40.61)	
	West	372 (30.77)	
**Age (years)**			
	21-24	182 (15.05)	
	25-29	233 (19.27)	
	30-34	248 (20.51)	
	35-39	189 (15.63)	
	40-44	146 (12.08)	
	45-49	88 (7.28)	
	≥50	123 (10.17)	
**Gender**			
	Male	545 (45.08)	
	Female	646 (53.43)	
	Other^a^	10 (0.83)	
	Prefer not to answer	8 (0.66)	
**Race**			
	American Indian or Alaska Native only	9 (0.74)	
	Asian or Asian American only	26 (2.15)	
	Black or African American only	45 (3.72)	
	Native Hawaiian or Pacific Islander only	4 (0.33)	
	White only	920 (76.09)	
	Other	42 (3.47)	
	Multiracial	134 (11.08)	
	Prefer not to answer	29 (2.39)	
**Ethnicity**			
	Hispanic, Latino, or of Spanish origin	129 (10.67)	
	Non-Hispanic, Latino, or of Spanish origin	1061 (87.76)	
	Prefer not to answer	19 (1.57)	
**Annual household income (US $)**			
	0-39,999	583 (48.22)	
	40,000-59,999	287 (23.74)	
	60,000-99,999	209 (17.29)	
	≥100,000	101 (8.35)	
	Prefer not to answer	29 (2.39)	
**e-Cigarette use (days per week)**			
	5	74 (6.12)	
	6	26 (2.15)	
	7	1109 (91.73)	
**Smoking status**			
	Nonsmoker^b^	711 (58.81)	
	Smoker^b^	498 (41.19)	

^a^Other gender includes transgender individuals, nonbinary individuals, and so on.

^b^Participants were considered non-smokers if they had not smoked in the past 30 days. Participants were considered smokers if they smoked cigarettes ≥1 time in the past 30 days.

### Availability of Data for Both Photos and Self-reports

The number of values excluded ranged from 4.88% (59/1209; device reusability) to 56.49% (683/1209; bottle size; Table 2). Photo values were excluded if device brand or model or liquid brand or flavor could not be identified from the photo (ie, no photo submitted, photo of the liquid was from the internet, poor quality of the photo, multiple devices or liquids in the photo, photo was of refillable or third-party pod or cartridge, or no match was found in Google searches) or the information could not be found on the internet after identifying the device brand or model or liquid brand or flavor. Self-reported values were excluded if the participant self-reported “I don’t know,” skip logic prevented participant from being asked the given question (eg, participants who indicated that their liquid was customized were not asked the brand of their liquid), or the self-reported response was not able to be cleaned owing to lack of clarity (eg, liquid brand reported as “local vape shop”). Of the 1209 participants, the resulting sample sizes for calculations ranged from 47 (3.89% for device model of disposable devices) to 1150 (95.12% for device reusability; Table 3).

**Table 2:** Excluded values for photos and self-report data by variable (N=1209).

Variables	Photo only, n (%)	Self-report only, n (%)				Both, n (%)				Total, n (%)
		Excluded for other reasons^a^	Excluded because question was not asked to participant		Excluded for other reasons^a^		Excluded because question was not asked to participant			
Device brand	42 (3.47)	289^b^ (23.90)		0 (0)		0 (0)		0 (0)		331 (27.38)
Device model	29 (2.40)	363 (30.02)		0 (0)		211 (17.45)		0 (0)		603 (49.88)
Device reusability	59 (4.88)	0 (0)		0 (0)		0 (0)		0 (0)		59 (4.88)
Device refillability	51 (4.22)	0 (0)		111 (9.18)		0 (0)		8 (0.66)		170 (14.06)
Device display	51 (4.22)	0 (0)		111 (9.18)		0 (0)		8 (0.66)		170 (14.06)
Adjustable power	51 (4.22)	0 (0)		111 (9.18)		0 (0)		8 (0.66)		170 (14.06)
Liquid brand	70 (5.79)	52 (4.3)		307 (25.39)		26 (2.15)		106 (8.77)		561 (46.40)
Liquid flavor	317 (26.22)	37 (3.06)		0 (0)		21 (1.74)		0 (0)		375 (31.02)
Nicotine concentration	321 (26.55)	50 (4.14)		0 (0)		63 (5.21)		0 (0)		434 (35.90)
Nicotine formulation	224 (18.53)	240 (19.85)		0 (0)		80 (6.62)		0 (0)		544 (45.00)
Liquid bottle size	167 (13.81)	72 (5.96)		352 (29.11)		37 (3.06)		55 (4.55)		683 (56.49)

^a^Other reasons include if the participant self-reported “I don’t know” or the self-reported response was not able to be cleaned owing to lack of clarity (eg, liquid brand reported as “local vape shop”).

^b^Includes records that were excluded because the brand was JUUL, Vaporesso, or Voopoo (n=270).

**Table 3:** Sample size for calculations by liquid and device type.

Variables	Device types, n (%)					Liquid types, n (%)	
	Overall (n=1209)	DD^a^ (n=119)	RDD^b^ (n=288)	RDR^c^ (n=802)	Noncustomized (n=915)		Customized (n=294)
Device brand	878 (72.62)	108 (90.8)	141 (48.9)	629 (78.4)	645 (70.5)		233 (79.3)
Device model	606 (50.12)	47 (39.5)	57 (19.8)	502 (62.6)	447 (48.9)		159 (54.1)
Device reusability	1150 (95.12)	111 (93.3)	280 (97.2)	759 (94.6)	878 (95.9)		272 (92.5)
Device refillability	1039 (85.94)	N/A^d^	280 (97.2)	759 (94.6)	767 (83.8)		272 (92.5)
Device display	1039 (85.94)	N/A	280 (97.2)	759 (94.6)	767 (83.8)		272 (92.5)
Adjustable power	1039 (85.94)	N/A	280 (97.2)	759 (94.6)	767 (83.8)		272 (92.5)
Liquid brand	648 (53.59)	N/A	251 (87.2)	397 (49.5)	648 (70.8)		N/A
Liquid flavor	834 (68.98)	92 (77.3)	166 (57.6)	576 (71.8)	687 (75.1)		147 (50)
Nicotine concentration	775 (64.10)	85 (71.4)	137 (47.6)	553 (68.9)	602 (65.8)		173 (58.8)
Nicotine formulation	665 (55)	61 (51.3)	106 (36.8)	498 (62.1)	519 (56.7)		146 (49.7)
Liquid bottle size	526 (43.51)	N/A	N/A	526 (65.6)	382 (41.7)		145 (49.3)

^a^DD: disposable devices.

^b^RDD: reusable devices with disposable pods or cartridges.

^c^RDR: reusable devices with refillable pods, cartridges, or tanks.

^d^N/A: not applicable.

### Agreement for Device and Liquid Characteristics Between Photos and Self-reports

Percentage agreement was high (≥80%) between photos and self-reports for device reusability, adjustable power, device display, device refillability, and liquid brand (Table 4). Very high agreement (≥91%) was also observed for device brand for all device and liquid types except disposable devices (75%). Substantial agreement (61%-80%) was found for device model for disposable devices and refillable devices; however, agreement was very high (91.2%) for reusable devices with disposable pods or cartridges, though the sample size for this calculation was limited (57/1209, 4.71%). Percentage agreement was high for liquid flavor, though reusable devices with disposable pods or cartridges had lower agreement (79.5%) than other device types (≥91.3%). Moderate to substantial agreement was found for nicotine concentration across device and liquid types (56.2% for customized liquids to 69% for refillable devices); however, this agreement was lower than that for other variables. Percentage agreement varied widely for nicotine formulation (58.5% for reusable devices with disposable pods or cartridges to 93.6% for refillable devices), though it was generally high. Substantial agreement was also found for bottle size overall (74.3%), though agreement was low for customized liquids (64.6%).

These results were largely supported by Krippendorff *α* calculations; however, agreement based on Krippendorff *α* was lower than the percentage agreement for several variables (Table 5). Results from the ICC calculations for nicotine concentration and bottle size (Table 6) show lower agreement for these variables than the results of the percentage agreement calculations.

**Table 4:** Results of percentage agreement calculations by liquid and device type.

Variables	Device types (%)					Liquid types (%)	
	Overall	DD^a^	RDD^b^	RDR^c^	Noncustomized		Customized
Device brand	92	75	97.9	93.6	91.3		94
Device model	72.6	74.5	91.2	70.3	72.5		73
Device reusability	98.8	91	99.3	99.7	98.4		100
Device refillability	96.7	N/A^d^	96.8	96.7	96.3		97.8
Device display	92.9	N/A	92.9	92.9	92.8		93
Adjustable power	93.9	N/A	97.1	92.8	95		90.8
Liquid brand	86.7	N/A	96	80.9	86.7		N/A
Liquid flavor	89.9	91.3	79.5	92.7	89.4		92.6
Nicotine concentration	66.3	65.9	56.2	69	68.1		60.1
Nicotine formulation	86.2	73.8	58.5	93.6	85		90.4
Liquid bottle size	74.3	N/A	N/A	74.3	78		64.6

^a^DD: disposable devices.

^b^RDD: reusable devices with disposable pods or cartridges.

^c^RDR: reusable devices with refillable pods, cartridges, or tanks.

^d^N/A: not applicable.

**Table 5:** Results of Krippendorff α calculations by liquid and device type.

Variables	Device types (Krippendorff *α*)					Liquid types (Krippendorff *α*)	
	Overall	DD^a^	RDD^b^	RDR^c^	Noncustomized		Customized
Device reusability	.93	−0.04	0	0	.93		1
Device refillability	.92	N/A^d^	−0.01	−0.02	.92		−0.01
Device display	.86	N/A	.13	.84	.85		.83
Adjustable power	.88	N/A	.32	.82	.90		.74
Liquid flavor	.78	.61	.66	.69	.79		.45
Nicotine formulation	.72	−0.14	−0.18	.86	.70		.76

^a^DD: disposable devices.

^b^RDD: reusable devices with disposable pods or cartridges.

^c^RDR: reusable devices with refillable pods, cartridges, or tanks.

^d^N/A: not applicable.

**Table 6:** Results of intraclass correlation coefficient calculations by liquid and device type.

Variables	Device types, estimate (95% CI)					Liquid types, estimate (95% CI)	
	Overall	DD^a^	RDD^b^	RDR^c^	Noncustomized		Customized
Nicotine concentration	0.21 (0.14 to 0.27)	0.01 (−0.14 to 0.18)	0.04 (−0.12 to 0.20)	0.18 (0.10 to 0.26)	0.20 (0.13 to 0.28)		0.16 (0.02 to 0.30)
Liquid container size	0.38 (0.30 to 0.45)	N/A^d^	N/A	0.38 (0.30 to 0.45)	0.35 (0.26 to 0.44)		0.47 (0.33 to 0.59)

^a^DD: disposable devices.

^b^RDD: reusable devices with disposable pods or cartridges.

^c^RDR: reusable devices with refillable pods, cartridges, or tanks.

^d^N/A: not applicable.

## Discussion

### Principal Findings

Although we found substantial to almost perfect agreement between photos and self-report for all variables measured in this study, agreement and ICC for nicotine concentration was substantially lower than those for other variables assessed. As previous research has identified [29], this may be a result of participants’ poor understanding of nicotine concentration labeling and particularly the differences in the units of reported concentrations (mg/mL vs percentage); some users reported JUUL pods at a concentration of 5 mg/mL, though JUUL sells pods at only 35 mg/mL (3%) or 59 mg/mL (5%) [23].

In addition, we found the lowest agreement for device brand and model for participants who were using disposable devices and the highest agreement for those using reusable devices with disposable pods or cartridges. Despite finding the highest agreement for device brand and model among users of reusable devices with disposable pods or cartridges, agreement for liquid flavor was lowest among these users. These differences may be owing to low knowledge of these characteristics among users of certain device types or inaccurate or incomplete information about these characteristics on websites for certain device types. The wide variation in agreement for nicotine formulation across device types suggests a need for future research into how to evaluate the nicotine formulation of a liquid and how to ensure adequate labeling of nicotine formulation, so that participants’ self-reports can be more accurate. Given that the lowest agreement was observed in users of disposable devices and reusable devices with disposable pods or cartridges, it may be that users of nonrefillable devices tend to be less informed about the nicotine formulation of their liquid, possibly owing to poor labeling or lack of concern about nicotine formulation. In addition, these may be a result of challenges in finding accurate information about nicotine formulation for disposable devices and reusable devices with disposable pods or cartridges on websites. Customized liquids presented a unique challenge in assessing bottle size, with lower agreement than noncustomized liquids. This is likely because of challenges in coding the photos of customized liquids, as users sometimes refill bottles obtained from previous purchases of brand-name liquids but may be reporting the quantity of refill rather than the actual size of the bottle. It is also possible that users of customized liquids have submitted photos of a noncustomized liquid owing to concerns that we will be unable to use data from an unlabeled customized liquid bottle.

Krippendorff *α* varied from the percentage agreement for certain variables, which is likely owing to differences in the prevalence of certain characteristics [26,27]. For example, the prevalence of reusable devices with disposable pods or cartridges in our sample with device displays (24/1209, 1.99% for self-report; 2/1209, 0.17% for photo) was low; Krippendorff *α* can be affected by extreme values of prevalence for a given measure [26,27]. In addition, results of the ICC calculations were lower than percentage agreement for nicotine concentration and bottle size. As ICC also accounts for the magnitude of the differences between values that disagree, this suggests that, when the self-report and photo values for nicotine concentration and bottle size disagree, the magnitude of the difference between the values is relatively large.

Disagreement between self-reports and photos may also be caused by inaccuracies in website data used to code photos (information about device and liquid characteristics are sometimes inconsistent across websites), user modifications of devices in manners inconsistent with manufacturer intended use of the product (eg, some consumers refill JUUL pods, which are intended to be disposable [30], with other liquids) [31], or submission of photos that are not a participant’s most used device or liquid. These issues present unique challenges in understanding e-cigarette device and liquid characteristics and warrant future research into understanding the prevalence of these issues and opportunities for potential solutions.

It is also important to note that the number of excluded values differ between self-reports and photos. This is an important consideration in deciding on an approach as missing values can reduce the sample size or lead to a requirement for more resources for recruitment to obtain a sufficient sample size. When discounting self-reported values that were excluded owing to the skip logic of the survey, self-reports have more usable data for all variables except device model and nicotine formulation. Values were excluded based on the skip logic in our survey only to avoid asking unnecessary questions or questions to which users are unlikely to provide reliable responses. Users of disposable devices (119/1209, 9.84%) were not asked about their device refillability, adjustable power, presence of a device display, liquid brand, or liquid bottle size; users of reusable devices with disposable pods or cartridges (288/1209, 23.82%) were not asked about their liquid container size; and users of customized liquids (294/1209, 24.32%) were not asked the brand of their liquid. Although both self-reports and photos may produce fairly accurate results for device model and nicotine formulation, as indicated by the generally high agreement for these variables, photos may ultimately be superior to self-reports for device model and nicotine formulation because they capture more usable data.

The results outlined in this study provide valuable information about assessing several characteristics of e-cigarette devices and liquids. The agreement between photos and self-reports was substantial to very high for all variables included in this study except nicotine concentration. In addition, photos may provide more usable data for device model and nicotine formulation, whereas self-reports may provide more usable data for the remaining variables: device brand, device reusability, adjustable power, device display, device refillability, liquid brand, liquid flavor, nicotine concentration, and liquid bottle size (for users of refillable devices). However, using both of these approaches in tandem may allow for higher data quantity and quality, as values missing from one approach can be supplemented using the other (ie, values missing from photo data may be filled in using data from self-reports and vice versa) and data can be cross-checked between the 2 approaches.

### Strengths and Limitations

The strengths of this study include the large sample size, which allows for analyses of subgroups of participants such as users of various device types. In addition, participants were not aware that they would be required to submit photos for the survey until after the self-report questions had been answered. Therefore, it is unlikely that the agreement data were affected as a result of participants being more accurate in their self-report owing to knowledge of the photo-uploading part of the study. However, the reverse may not be true; it is possible that some participants were more likely to upload photos of the device or liquid they self-reported because they had already reported information about the device or liquid.

Although the questions included in the survey were selected from or based on previously validated questions (eg, PhenX Toolkit) or established surveys (eg, Population Assessment of Tobacco and Health and International Tobacco Control survey) where possible, novel questions were created if a given characteristic had not been previously assessed or validated (eg, device reusability). In addition, the rigorous data review procedures in this study (eg, eliminating participants with very low knowledge of e-cigarette devices or liquids; photos from the internet; or photos with a brand that did not match the self-reported brand for JUUL, Voopoo, and Vaporesso and requesting a utility bill for participants who failed an initial identity authentication) may have resulted in a sample with a larger proportion of highly conscientious participants, and thus, higher agreement between self-reports and photos than we may otherwise have seen. JUUL, Voopoo, and Vaporesso were excluded from device brand analyses. As these were provided in the question prompt for brand as examples, bots and other invalid submissions frequently listed these as brand and submitted photos of other devices. These submissions were excluded from our analyses and, therefore, any valid self-reported JUUL, Voopoo, and Vaporesso submissions would be a match for photo brand. Therefore, the results for brand calculations cannot be extended to include these 3 brands. Results should be interpreted carefully with the understanding that “I don’t know” or missing responses were excluded from the percentage agreement, Krippendorff *α*, and ICC calculations; therefore, the level of agreement found in this study applies only to complete responses. Self-reported bottle size was assessed only among users of refillable devices owing to concerns that participants may not reliably report this information; therefore, the results for this variable may not extend to users of disposable devices or reusable devices with disposable pods or cartridges. Owing to time and resource constraints, our survey only included questions about and provided a photo submission option for participants’ most used device and liquid; it is possible that agreement between self-report and photo data may vary when asking about users’ alternative devices and liquids. In addition, only 11 characteristics were included in the analyses; results cannot be applied to other variables such as device wattage, voltage, or resistance. The distribution of our sample with respect to gender and race was similar to that of daily e-cigarette users in the nationally representative Tobacco Use Supplement to the Current Population Survey; however, participants in our study were relatively young and had low income. It is possible that participants from different backgrounds may report more or less consistently between self-reports and photos. Totally, 89.99% (1088/1209) of our sample used e-cigarettes on 7 days per week; this may have selected for more knowledgeable e-cigarette users. Percentage agreement may be lower in a population that uses e-cigarettes less frequently as compared with this study. As we decided not to establish a gold standard between self-report and photo data, we cannot establish which method yielded more accurate results. The time requirements for collecting and processing data for each method also was not considered in this study.

### Conclusions

e-Cigarette device and liquid data from both self-reports and user-submitted photos can present challenges. The high agreement between self-reports and photos suggests that these 2 methods yield data of similar accuracy for several variables: device brand, device model, device reusability, presence of adjustable wattage, presence of a display, device refillability, liquid brand, liquid flavor, and liquid bottle size. Self-reports provided a higher quantity of data than photos for all these variables except device model and nicotine formulation, for which photos provided a higher quantity of data. Although self-reports may be sufficient in certain studies and for specific variables, using these 2 approaches in tandem presents an opportunity to optimize the quality and quantity of data as it allows data to be cross-checked between 2 sources and provides an additional source when data are missing from one source. Further research is needed to understand how to assess nicotine concentration and other variables not included in these analyses (eg, wattage and resistance), how consistently and accurately the users of disposable devices and reusable devices with disposable pods or cartridges report on their liquid bottle size, the time and resource requirements for successful implementation of each of these 2 approaches (ie, photos and self-reports), and potential new innovative techniques for assessing e-cigarette device and liquid characteristics (eg, video recordings and daily e-cigarette use diaries).

## Abbreviations

ICC: intraclass correlation coefficient
REDCap: Research Electronic Data Capture
